# Role of narrative medicine-based education in cultivating empathy in residents

**DOI:** 10.1186/s12909-023-04096-5

**Published:** 2023-02-21

**Authors:** Jianhua Zhao, Ouyang Xiantao, Qiong Li, Hao Liu, Fan Wang, Qing Li, Zhixiu Xu, Sibei Ji, Shuangzhu Yue

**Affiliations:** 1 Henan Joint International Research Laboratory of Neurorestoratology for Senile Dementia, Henan Key Laboratory of Neurorestoratology, Department of Neurology, First Affiliated Hospital of Xinxiang Medical University, 453100 Weihui, Xinxiang, Henan China; 2 Management Institute of Xinxiang Medical University, 453003 Xinxiang, Henan China; 3 Resident Standardized Training Center, the First Affiliated Hospital of Xinxiang Medical University, 453100 Weihui, Xinxiang, Henan China

**Keywords:** Narrative medicine, Residents, Humanities, Empathy, Standardized resident training, Parallel medical records

## Abstract

**Objective:**

To explore the role of narrative medicine-based education in standardized empathy training for residents.

**Methods:**

Among the 2018–2020 residents at the First Affiliated Hospital of Xinxiang Medical University, 230 receiving neurology training were enrolled in this study and randomly divided into study and control groups. The study group received narrative medicine-based education and standardized routine resident training. The Jefferson Scale of Empathy–Medical Student version (JSE–MS) was used to evaluate empathy in the study group, and the neurological professional knowledge test scores of the two groups were also compared.

**Results:**

In the study group, the empathy score was higher than the preteaching score (*P* < 0.01). The neurological professional knowledge examination score was higher in the study group than in the control group, albeit not significantly.

**Conclusion:**

The addition of narrative medicine-based education in standardized training improved empathy and may have improved the professional knowledge of neurology residents.

Residents shoulder the responsibility of primary medical security in China. Thus, improvements in the humanistic qualities of residents play a positive role in reshaping harmonious doctor‒patient relationships, thereby improving people’s health index scores. In the residency training stage, the main training content includes medical theoretical knowledge, practical abilities, medical ethics and styles, relevant laws and policies, and communication abilities. However, in actual work, some training results may not be satisfactory, such as a lack of medical humanistic literacy, which directly or indirectly leads to biased medical education programs that focus only on medical technology itself while ignoring the patient as a whole social person. Moreover, the existing training methods in China lack objectivity and are more in line with the actual situation in China.

The concept of “narrative medicine-based education” was introduced in China 10 years ago, and the three core elements are attention, representation and affiliation. However, there is a lack of narrative medicine-based education at the clinical skills training stage, so it is expected that strengthening narrative medicine-based education will improve residents’ humanistic abilities. As an embodiment of the essential quality of doctor‒patient communication and the humanistic spirit, empathy is a professional quality that is essential in medical personnel [1]. Narrative medicine helps to develop clinicians’ narrative abilities and improves their empathy, affinity, professionalism, and professional spirit towards patients [2]. This medical concept regarding humanistic care has been widely accepted and practised in China in recent years, especially in the standardized training of residents [3]. The narrative medicine-based education model gives more attention to humanistic qualities and truly incorporates the idea of being “people-oriented”. At present, in the early stage of residency training, more attention is given to theoretical teaching. Narrative medicine-based education can better compensate for the above shortcomings and improve the current medical education model. Moreover, narrative medicine-based education can improve doctors’ professional qualities [4]. Therefore, in this study, narrative medicine-based education was applied to clinical training to explore its impact on residents’ empathy and academic performance.

The residency training system in China is different from that in other Western countries. A qualification examination before standardized residency training is not needed in China, and the training period is three years. However, at the same time, there are similarities among training systems. For example, residents have different specialties for which they must perform different rotations. The “residents” covered in this study are those who need to systematically learn basic medical skills in primary community hospitals. Such residents can obtain a practising physician qualification certificate and register only after their first year of training. The participants included in this study were in the clinical standardized resident training stage and were currently training in neurology.

## Subjects and methods

### Study participants

A total of 230 residents who underwent standardized resident training in the Department of Neurology of the First Affiliated Hospital of Xinxiang Medical University from December 2018 to December 2020 were randomly divided into the study group (n = 116) and the control group (n = 114).

### Teaching method

The control group received standardized training in clinical teaching, and the study group received standardized training with the addition of narrative medicine teaching. The main goal of standardized clinical neurology training was for residents to master the diagnosis and treatment of common neurological diseases and routine neurological practices. The teaching methods were mainly classroom lectures and in-ward operation demonstrations. The duration of the training and study was 2 months. Within 2 months of receiving the training, the study group also received narrative medicine education training, with teaching methods including classroom lectures, discussions, films, readings, and the analysis of narrative medicine. The participants in the study were residents receiving standardized residency training during the 2018–2020 academic year. The age range was from 24 to 27 years. All the subjects provided informed consent to participate in the study. The residents were trained in neurology for 2 months, with each session lasting 1 h/week. Moreover, the study group was required to complete a parallel medical record every week and read and discuss it in class. The teachers providing the training were attending physicians or above. The teachers who provided the narrative medicine education training had received adequate narrative medicine education. Teachers with an adequate narrative medicine-based education mainly referred to (1) those who were attending doctors or above and had teaching experience at medical universities; (2) those who were trained by international experts; and (3) those with publications in narrative medicine. Upon completion of the course, the instructor awarded the relevant completion certificate.

### Evaluation of the teaching effect

#### Compositional ability

The level of empathy in the study group was evaluated using the Chinese version of the Jefferson Empathy Scale (the Jefferson Scale of Physician Empathy–Student, JSPE-S) before and after training. The scale is based on empathy in medical education and practice and evaluates empathy based on doctors’ medical education and patient care [5]. Scores are calculated by using a 7-point Likert scale with 10 positive scores and 10 negative scores; total scores range from 20to 140, and the higher the score, the greater the level of empathy was. The Chinese version of the JSPE-S has good reliability and is suitable for Chinese residents [6].

#### Assessment of neurology expertise

After the end of all courses, the study and control groups were tested at the Department of Neurology. The specific scoring method was as follows: (1) Theoretical knowledge, assigned 40 points; (2) skill operation, assigned 30 points; and (3) clinical management, case teaching and case discussion, assigned 30 points. The above 3 items compose the contents of the professional ability assessment. The total possible score is 100. The above content is based on the questionnaire format.

### Statistical analysis

SPSS 26.0 was used for statistical analysis of the relevant data. Measurement data are expressed as the mean ± standard deviation (mean ± SD), and all residents’ empathy scores were measured by the paired t test; *P* values < 0.05 indicated statistically significant differences.

## Results

### Comparison of empathy before and after training

The 116 residents in the study group actively participated in the narrative medicine education course and provided parallel medical records. The empathy scores of the residents before and after narrative medicine education were 110.6 ± 12.1 and 122.6 ± 9.0, respectively, and the difference was statistically significant (*t*=-4.399, *P* < 0.05).

### Comparison of professional knowledge achievements in the neurology department

All 230 residents completed the standardized training requirements for residents in our hospital. All trainees participated in teaching activities, such as the clinical management of patients, teaching ward rounds, and discussion of difficult cases, and received training in basic skills for a prescribed number of diseases. The residents recorded the scores of the neurology group (study group: 74.33 ± 9.6; control group: 69.1 ± 8.4), which were statistically different (t=-3.485, *P* < 0.05).

## Conclusion

Doctors with certain narrative abilities practice clinical narrative medicine. Narrative ability is defined as the ability of doctors to understand, absorb, explain, and be moved by the real story of the disease [7]. Narrative medicine education focuses on active listening by doctors, the personalities, experiences, stories and questions of patients and doctors’ explanations to patients regarding the various risk factors of treatment options and diseases in layman’s terms [8]. At the same time, attention should be given to more nonverbal communication and perceptual interactions in medical activities, which can help generate more empathy [9]. Such communication between doctors and patients increases patients’ compliance and doctors’ humanistic qualities [10].

The two main findings of this study were as follows. First, narrative medicine-based education significantly improved residents’ empathy, and the empathy scores of the conventional practitioners were significantly improved after narrative medicine-based training compared to those before training, which was consistent with previous findings [11]. In particular, narrative medicine-based training plays a positive role in the cultivation of empathy [12]. This showed that the combination of narrative medicine and the traditional standardized training for residents could achieve better teaching results and enhance residents’ technical knowledge and humanistic qualities. The underlying reason may be that doctors are involved in the process of narrative medicine teaching by reading narrative medicine-related books and watching related movies, expanding medical humanities knowledge by cultivating “reading” abilities, reading patients as if reading a text, and noting their families’ psychological state and emotional demands. The doctor’s own perceptions can provide a better understanding of the patient’s needs and better support [13]. In addition, parallel reflective writing is involved when residents and patients participate in a role exchange, i.e., playing sick to understand the patient’s perspective of their problems, thereby allowing medical students to introspect and increase and achieve empathy with their patients. Moreover, a longitudinal prospective study showed that the structure of the JSE results were different for students in different grades or with different cultural backgrounds, which suggests that the use of the JSE may have certain shortcomings and cross-cultural differences [14, 15]. These drawbacks may require the use of additional scales.

Second, residents who received narrative medicine-based training showed markedly improved professional knowledge. LaRocque demonstrated that narrative medicine-based education improved residents’ performance in objective and structured clinical examinations (OSCEs) [16]. The high level of theoretical knowledge of the study group might be due to the integration of narrative medicine-based education and increased attention towards patients in clinical practice. Based on the stories of patients in the clinical setting, listening, thoughts, and parallel medical records could improve doctors’ understanding of the disease history and characteristics while in contact with the patient, improve disease diagnosis, and reduce doctor burnout through empathy, trust, and partnership, making residents more willing to spend more time in medical training [17, 18]. Increased burnout and fatigue among physicians can lead to additional negative outcomes. Moreover, empathy is controllable and can be cultivated through deliberate training [19]. Increasing ’residents’ proprioceptive abilities as well as philosophical discussions also contributes to the development of empathy. Therefore, it is important to explore effective and constructive ways to cultivate empathy [20]. Although the level of empathy is related to personality traits, some inherent personality traits can be improved through practical training. In this study, narrative medicine-based education training was conducted for 2 months, and the results showed that short periods of training increased the residents’ empathy scores. Another study showed that empathy gradually declined as students progressed, which may be related to the accumulation of fatigue in practice, comparisons between classmates, and insecurity [21]. The standardized training stage is an important period for medical residents to gradually shift from the classroom to clinical practice. Especially in the early stage of training, residents have sufficient experience in classroom education and have a psychological interest in clinical practice. Residents are more receptive and less fatigued in early stages of training, and narrative medicine-based education may be more efficient at this stage.

Narrative medicine-based education is a replicative and effective teaching method. Almost all modes follow the three steps of reading, reflection and response, especially the last step. Through the above three points, it is possible to cultivate residents’ abilities to process large amounts of clinical information. Thus, the impact of personal experiences and biases on disease diagnosis is reduced [22].

In summary, the combination of narrative medicine-based education with standardized clinical training has better teaching results than standard training alone. Narrative medicine-based education can significantly improve the humanistic qualities and empathy of doctors undergoing regular training, thereby improving their academic performance. Furthermore, narrative medicine-based education is often patient-centred and emphasizes humane patient conditions, which requires relieving and even eliminating the patients’ symptoms and pain and pursuing harmony and health [23]. Thus, narrative medicine-based education can improve individual residents’ medical and humanistic literacy and enhance doctor‒patient harmony [24]. These findings indicated that narrative medicine-based education plays a vital role in the standardized training system of resident physicians.

## Data Availability

All data generated or analysed during this study are included in this published article.
